# Primary diffuse large B-cell lymphoma of bone in adults: A SEER population-based study

**DOI:** 10.1097/MD.0000000000040071

**Published:** 2024-10-25

**Authors:** Jing Li, Xiangpeng Li, Tong Gao, Changkai Zhou, Qie Guo, Donghua Liu

**Affiliations:** a Department of Clinical Pharmacy, Affiliated Hospital of Qingdao University, Qingdao, Shandong, China.

**Keywords:** disease-specific survival, nomogram, overall survival, primary bone diffuse large B-cell lymphoma, primary bone lymphoma

## Abstract

Primary diffuse large B-cell lymphoma of the bone (PB-DLBCL) is an extremely rare type of extra-nodal lymphoma. The clinical characteristics, management, and survival outcomes of adult PB-DLBCL patients remain poorly defined. To explore the clinical manifestations, staging, therapeutic options, prognostic factors and outcomes of adult patients with PB-DLBCL and to create a model to predict survival outcomes. Data of adult PB-DLBCL patients were obtained from the Surveillance, Epidemiology, and End Results (SEER) Program 18 registries database from 2000 to 2018. The Kaplan–Meier survival analysis was conducted to calculate survival rates. Univariate Cox regression, best subset selection (BESS), and least absolute shrinkage and selection operator (LASSO), followed by backward stepwise multivariable Cox regression, were used to construct the nomogram. The nomograms were evaluated using the concordance index (C-index), calibration curves and decision curve analysis (DCA). Diffuse large B-cell lymphoma (DLBCL) (67.51%) was the most frequent type of primary bone lymphoma. The most involved sites were the spine and lower-limb long bones. For the whole cohort, the 3-, 5-, 10- and 15-year overall survival (OS) rates were 74.9%, 70.5%, 60.0%, and 49.9%, and corresponding disease-specific survival (DSS) rates were 79.7%, 77.8%, 75.1%, and 71.4%, respectively. For OS, age, Ann Arbor stage, primary site and therapy were confirmed as final factors to develop the nomogram in adult PB-DLBCL patients, whereas for DSS, Age, marital status, Ann Arbor stage, number of bone lesions, therapy and year of diagnosis were confirmed as final factors in developing the nomogram. The nomograms demonstrated good accuracy and clinical utility. Established nomograms can accurately predict the survival of patients with PB-DLBCL and help clinicians optimize treatment.

## 1. Introduction

Primary bone lymphoma (PBL) is a rare lymphoid malignancy.^[1]^ In 1939, PBL was first reported by Parker and Jackson; and was diagnosed as “primary reticulum cell sarcoma of bone.” PBL has been characterized by the World Health Organization (WHO) Classification as a primary skeletal malignancy, with 1 or multiple bone lesions, and without other extra-nodal or lymph-node involvement.^[2]^ It was officially named as PBL in 2013 by WHO. PBL represents 7% of bone malignancy, 4% to 5% of all extra-nodal lymphomas and ~1% of non-Hodgkin lymphoma.^[2]^ The main clinical manifestations of PBL are bony pain, followed by mass/swelling and pathological fracture.^[3]^ Patients with spinal or cranial lesions may have corresponding symptoms of nerve compression, and only a small number of patients experience systemic symptoms like fever, night sweats, or weight loss.^[4]^ Stage of PBL is usually defined according to the Ann Arbor staging system: stage i.e. = single localized bone lesions; stage IIE = single bony lesion with involvement of regional lymph nodes; stage IV = multifocal disease in a single bone or lesions in multiple bones in a disease exclusively limited to the skeleton (without lymph nodal or visceral disease).^[5]^ Although PBL can occur in all bones, the most frequent sites are the axial skeletons (including vertebral column, rib, sternum, etc).^[6,7]^PBL can occur at any age, but the peak of the onset is usually 40 to 60 years old.^[8]^ The incidence of PBL was higher in males than females in previous studies,^[3,8]^ but upon SEER-based population study, Liu et al indicated that no sex bias was observed for PBL.^[7]^

Diffuse large B-cell lymphoma (DLBCL) is the most frequent pathological type of PBL, which accounts more than 60% in previous studies.^[5,7]^ In primary diffuse large B-cell lymphoma of bone (PB-DLBCL) patients, the most common sites were the spine, which was consistent with the PBL studies.^[9,10]^ In a study of adult Jordanian patients with PB-DLBCL, all 12 patients reported pain over the site of the involved bone and 3 patients had masses.^[9]^ Pathological fractures was present in all patients with vertebral involvement.^[9]^ Due to the rarity of PB-DLBCL, there is no uniform standard for the current treatment scheme. The mainstays of therapy are radiotherapy, chemotherapy and surgery. Currently, the approved standard treatment for PB-DLBCL patients are cyclophosphamide, doxorubicin, vincristine, and prednisone (CHOP) and CHOP-like regimens, whether followed by radiotherapy or not.^[4]^ Rituximab in addition to chemotherapy for PB-DLCBL, started after 2000. Although rituximab in combination with chemotherapy showed significant improvements in the prognosis of patients with PB-DLCBL,^[11]^ the risk-benefit profile of radiotherapy as a consolidative therapy remains controversial.^[12,13]^ Surgical intervention was used when the patient had nerve compression, pathological fracture, or biopsies.

Due to the rarity of PB-DLBCL, previous studies were few and mainly derived from a single-center with a limited number of cases. As a result, the description of its clinical characteristics, management and prognosis remains unclear. Numerous factors can influence the survival outcome of patients with PB-DLBCL, of which age is an important factors. PBL in pediatric patients is considered to be markedly different from its counterpart in adults.^[14,15]^ Therefore, our study only included adult patients with PB-DLCBL above the age of 18. We analyzed 1603 adult patients with PB-DLBCL who were registered in the Surveillance, Epidemiology, and End Results (SEER) database 8. Predictive nomograms were established to predict overall survival (OS) and disease-specific survival (DSS) of patients with PB-DLBCL and help clinicians make scientific decisions. Furthermore, we assessed the calibration, discrimination and clinical utility of the models.

## 2. Materials and methods

### 2.1. Patients and methods

This study included 1603 adult patients with PB-DLBCL who were extracted from the publicly available SEER 18 database. Univariate Cox regression, best subset selection (BESS), and least absolute shrinkage and selection operator (LASSO), separately followed by backward stepwise multivariable Cox regression, were conducted to determine the independent prognostic factors of OS and DSS, and then predictive nomograms were created.

PBL patients were extracted from the publicly available SEER database from 2000 to 2018 (http://www.seer.cancer.gov), which contained records collected from 18 SEER cancer registries. Approximately 35% of the United States population is covered in SEER database, which annually provide information regarding of patient demographics, histologic type, primary anatomic sites, tumor stage, treatment and follow-up. Relevant data were extracted using SEER^*^ Stat 8.4.0. As rituximab was added to chemotherapy for PB-DLCBL after 2000, only patients diagnosed with PB-DLCBL after 2000 were enrolled in this study. PBL in pediatric patients is considered to be markedly different from its counterpart in adults.^[14,15]^ Therefore, our study only included adult patients with PB-DLCBL above the age of 18 years. The SEER program is a public cancer database, and patient information was completely anonymous. Therefore, the need for institutional review board approval and informed consent was waived.

**Figure 1.**
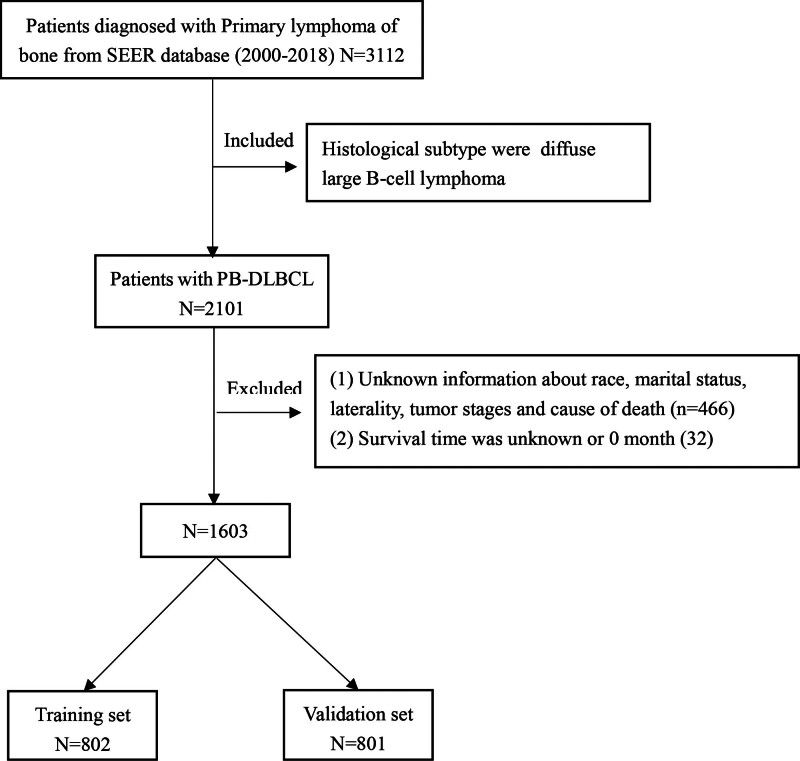
Diagrammatic flow of patient selection.

A flowchart of patient selection is shown in Figure 1. There of 3112 PBL were identified from the SEER database. Among them, 1603 patients with PB-DLBCL had complete clinical and follow-up information for analysis. They were randomly divided into 2 groups: training set (n = 802) and validation set (n = 801). The inclusion for the subjects were as follows: patients aged ≥ 18-years old; primary site of bone (SEER site code: C40.0–C41.9); lymphoma (SEER histology codes: 9590–9729); and malignancy (SEER behavior code: 3). Exclusion criteria were as follows: patients with missing data (stage, race, marital status, laterality, follow-up, etc); multiple bone lesions with concomitant lymphadenopathy (wherein biopsy was from node); single bone lesion with regional lymphadenopathy (wherein biopsy was from node); disseminated supra and infra-diaphragmatic nodal disease with concomitant bone lesions (wherein biopsy was from lymph-node); disseminated extra-nodal disease with bone lesions, wherein biopsy was from extra-nodal site (except bone).

### 2.2. Statistical analysis

The following variables were all recorded: age, race, sex, marital status, stage, primary site, laterality, number of bone lesions, surgery, radiotherapy, chemotherapy, year of diagnosis, survival time, cause of death, and vital status. Data are presented as frequencies (percentages) for categorical variables. Age showed non-normal distribution and was expressed as median (25th, 75th percentile). In the training set, univariate Cox hazard analysis, BESS, and LASSO regression were performed to identify the significant prognostic factors. In the training set, univariate Cox, BESS, and LASSO regression analyses were performed to identify significant prognostic factors. Then, backward stepwise multivariate Cox regression was used in each model separately to determine the independent significant prognostic factors. The Akaike information criterion (AIC) and receiver operating characteristic (ROC) curves were used to determine the optimal model. Predictive nomograms were developed by integrating the independent prognostic factors. The survival of patients was plotted by Kaplan–Meier survival curve. The accuracy of the nomogram was assessed based on concordance index (C-index) and calibration curves.^[16]^ The clinical net-benefit was evaluated by decision curve analysis (DCA). The R software (version 4.1.2) was used for data analysis (http://www.r-project.org/). These tests were bilateral, and *P* < .05 was defined as statistically significant.

## 3. Results

### 3.1. Patient baseline characteristics

The information of 3112 adult patients with PBL was extracted from the SEER18 database (2000–2018). Table 1 shows the histological types of PBL. DLBCL was the most frequent pathological type of PBL (67.51%). Overall 1603 adult patients with PB-DLBCL were enrolled in our study. They were randomly divided into 2 groups: training set (n = 802) and validation set (n = 801). The median patient age was 63 years (interquartile range, 47.5–74), and 53.6% were male. Whites account for 88.8%. Most of the patients (79.7%) were married. According to Ann Arbor staging, patients were categorized as the following: stage I (n = 867, 54.1%), stage II (n = 198, 12.4%), stage III (n = 32, 2.0%), and stage IV (n = 506, 31.5%). Table 2 shows the detailed distribution of the lesion site in PB-DLBCL. Of the bones involved, the vertebral column (n = 452, 28.2%) was the most common site, followed by the long bones of lower limb (n = 386, 24.08%). Most bone lesions occurred in the axial skeleton (n = 877, 54.7%), followed by the appendicular skeleton (n = 585, 36.5%) and the craniofacial skeleton (n = 141, 8.8%). The axial bones include the spine, pelvic bones, sacrum, coccyx, rib, sternum, clavicle, and associated joints. The appendicular bones include the short and long bones of the lower limbs, the short and long bones of the upper limbs and scapula, and the craniofacial bones include the mandible, skull, and face bones. Of all patients, 117 patients received radiation therapy as the sole therapy and 1366 patients (85.2%) underwent chemotherapy with or without radiotherapy. Only 349 (21.8%) received surgery.

**Table 1:** The distribution of histologic subtypes in PBL.

Histologic type (ICD-O-3)	Number	Percentage
9680: Diffuse large B-cell lymphoma (DLBCL), NOS	2101	67.51
9591: Non-Hodgkin lymphoma, NOS	343	11.02
9690: Follicular lymphoma	258	8.29
9590: Malignant lymphoma, NOS	90	2.89
9699: Extra-nodal marginal zone lymphoma of mucos-associated lymphoid tissue-MALT	67	2.15
9670: Malignant lymphoma, small B lymphocytic, NOS	41	1.32
9687: Burkitt lymphoma	35	1.12
9714: Anaplastic large cell lymphoma, ALK-positive	33	1.06
9671: Lymphoplasmacytic lymphoma	32	1.03
9650: Classical Hodgkin lymphoma	27	0.87
9684: Malig. lymphoma, large B, diffuse, immunoblastic (OBS 2012+)	17	0.55
9702: Peripheral T-cell lymphoma, NOS	17	0.55
9675: Malig lymphoma, mixed small & large cell, diffuse (OBS 2010+)	13	0.42
9673: Mantle cell lymphoma	10	0.32
9596: B-cell lymphoma, between diffuse large B and HL (composite HL and NHL)	6	0.19
9728: Precursor B-lymphoblastic lymphoma	5	0.16
9663: Nodular sclerosis classical Hodgkin lymphoma	4	0.13
9727: Blastic plasmacytoid dendritic cell neoplasm	3	0.10
9652: Mixed cellularity classical Hodgkin lymphoma	2	0.06
9688: T-cell histiocyte-rich large B-cell lymphoma	2	0.06
9719: Extra-nodal NK-/T-cell lymphoma, nasal type	2	0.06
9729: Precursor T--cell lymphoblastic lymphoma, NOS	2	0.06
9678: Primary effusion lymphoma	1	0.03
9665: Hodgkin lymphoma, nodular sclerosis, grade 1	1	0.03
Total patients with PBL	3112	100

ALK = anaplastic lymphoma kinase, HL = Hodgkin lymphoma, ICD = International classification of diseases, NHL = non-Hodgkin lymphoma, NK = natural killer (cell), NOS = not otherwise specified, PBL = primary bone lymphoma, PBL = primary bone lymphoma.

**Table 2:** The distribution of primary anatomic sites in PB-DLBCL.

Primary anatomic sites (ICD site code)	Number	Percentage
Vertebral column (C41.2)	452	28.20
Long bones of lower limb and associated joints (C40.2)	386	24.08
Pelvic bones, sacrum, coccyx and associated joints (C41.4)	213	13.29
Long bones of upper limb, scapula, and associated joints (C40.0)	164	10.23
Bone, NOS (C41.9)	129	8.05
Bones of skull and face and associated joints (C41.0)	87	5.42
Rib, sternum, clavicle and associated joints (C41.3)	66	4.12
Mandible (C41.1)	54	3.37
Short bones of lower limb and associated joints C (40.3)	24	1.50
Overlap bones, joints, and art. Cartilage (C41.8)	17	1.06
Bone of limb, NOS (C40.9)	6	0.37
Short bones of upper limb and associated joints (C40.1)	3	0.19
Overlap of bones, joints, and art. cartilage of limbs (C40.8)	2	0.12

ICD = International classification of diseases, NOS = not otherwise specified, PB-DLBCL = primary diffuse large B-cell lymphoma of bone.

**Figure 2.**
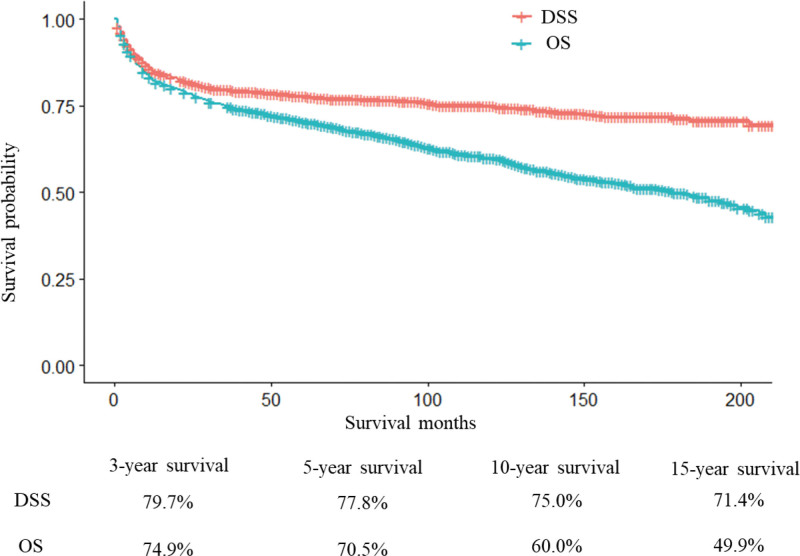
Kaplan–Meier curves of overall survival and disease-specific survival for adult patients with PB-DLBCL. DSS = disease-specific survival, OS = overall survival.

The median follow-up time was 107 months (interquartile range, 63–154). In total, 642 deaths occurred during the follow-up period. Among them, 385 patients died of PB-DLBCL and 257 died of other causes. During the 3-years follow-up period, 316 patients died of PB-DLBCL and 84 died of other causes. During the 15-years follow- up period, 383 patients died from PB-DLBCL and 247 died of other causes. The demographic information and patients characteristics were showed in Table 3. Figure 2 shows the Kaplan–Meier curves for OS and DSS in all patients. The 3-, 5-, 10-, and 15-year OS rates were 74.9%, 70.5%, 60.0%, and 49.9% and corresponding DSS rates were 79.7%, 77.8%, 75.1%, and 71.4%, respectively.

**Table 3:** Demographics and clinical characteristics for PB-DLBCL patients.

Variables	All patients (n = 1603)	Training set (n = 802)	Validation set (n = 801)	*P*
Age (yr)	63 (47.5,74)	63 (48,74)	63 (47,74)	.999
Race				
White	1411 (88.0%)	702 (87.5%)	709 (88.5%)	.532
Black	108 (6.7%)	53 (6.6%)	55 (6.9%)	
Other	84 (5.3%)	47 ((5.9%))	37 (4.6%)	
Sex				
Male	859 (53.6%)	435 (54.2%)	424 (52.9%)	.600
Female	744 (46.4%)	367 (45.8%)	377 (47.1%)	
Marital status				
Married	1278 (79.7%)	645 (80.4%)	633 (79.0%)	.486
Unmarried	325 (20.3%)	157 (19.6%)	168 (21.0%)	
Ann Arbor stage				
Stage I	867 (54.1%)	428 (53.4%)	439 (54.8%)	.836
Stage II	198 (12.4%)	98 (12.2%)	100 (12.5%)	
Stage III	32 (2.0%)	18 (2.2%)	14 (1.7%)	
Stage IV	506 (31.5%)	258 (32.2%)	248 (31.0%)	
Primary site				
Axial	877 (54.7%)	441 (55.0%)	436 (54.4%)	.400
Appendicular	585 (36.5%)	298 (37.2%)	287 (35.8%)	
Craniofacial	141 (8.8%)	63 (7.9%)	78 (9.7%)	
Laterality				
Bilateral, single primary	11 (0.7%)	7 (0.9%)	4 (0.5%)	.705
Left: origin of primary	391 (24.4)	192 (23.9%)	199 (24.8%)	
Right: origin of primary	359 (22.4%)	175 (21.8%)	184 (23.0%)	
Not a paired site	842 (52.5%)	428 (53.4%)	414 (51.7%)	
Number of bone lesions				
Single	1211 (75.5%)	611 (76.2%)	600 (74.9%)	.552
Multiple (⩾2)	392 (24.5%)	191 (23.8%)	201 (25.1%)	
Surgery				
No	1254 (78.2%)	621 (77.4%)	633 (79.0%)	.439
Yes	349 (21.8%)	181 (22.6%)	168 (21.0%)	
Therapy				
Chemotherapy alone	566 (35.3%)	279 (34.8%)	287 (35.8%)	.602
No therapy	120 (7.5%)	64 (8.0%)	56 (7.0%)	
Chemotherapy + radiation	800 (49.9%)	406 (50.6%)	394 (49.2%)	
Radiation alone	117 (7.3%)	53 (6.6%)	64 (8.0%)	
Overall survival				
Alive	961 (60.0%)	491 (61.2%)	470 (58.7%)	.298
Dead	642 (40.0%)	311 (38.8%)	331 (41.3%)	
Disease-specific survival				
Censored	1218 (76.0%)	616 (76.8%)	602 (75.2%)	.439
Dead	385 (24.0%)	186 (23.2%)	199 (24.8%)	
Year of diagnosis				
2000–2009	908 (56.6%)	464 (57.9%)	444 (55.4%)	.327
2010–2018	695 (43.4%)	338 (42.1%)	357 (44.6%)	

Note: Categorical variables are presented as percentages, and ages are presented as median with interquartile because of non-normal distribution.

### 3.2. Independent prognostic factors

**Figure 3.**
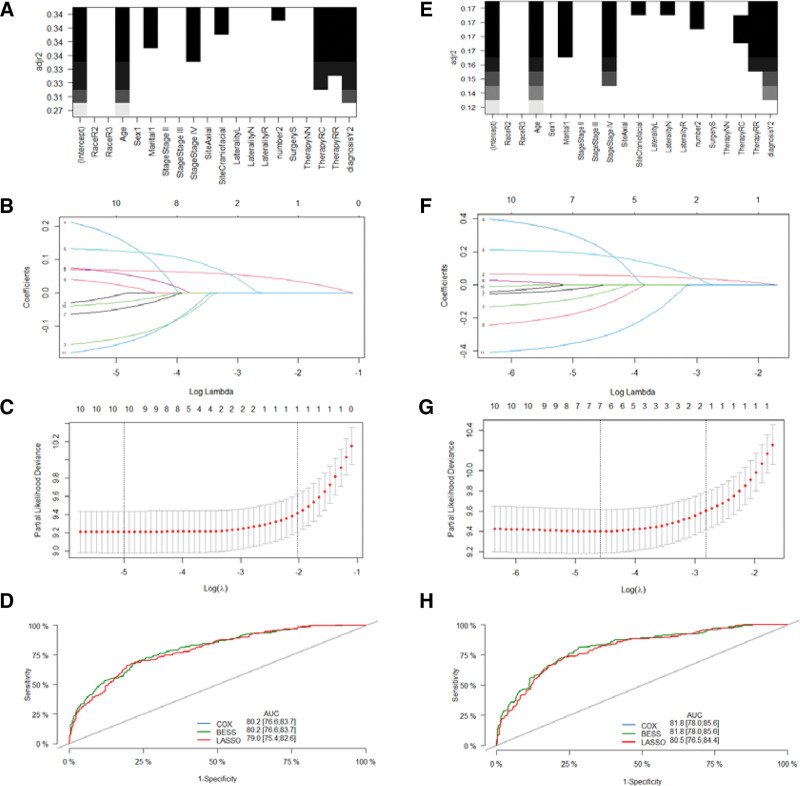
Variables screening and comparison among models in the training set (A–D) (OS) and (E–H) (DSS). (A and E) The best subset selection for variables screening; (B, C, F, and G) Least absolute shrinkage and selection operator regression for variables screening; (D and H) Comparison of ROC curves and AUC among COX, BESS and LASSO.

Univariate analyses revealed that age at diagnosis, marital status, Ann Arbor stage, primary site, number of bone lesions and therapy were associated with OS in the training set (*P* < .05, Table 4). In BESS, 7 variables (age at diagnosis, marital status, Ann Arbor stage, primary site, number of bone lesions, therapy and year of diagnosis) were selected based on the maximum value of the adjusted R2 (Fig. 3A). Using LASSO regression, we selected lambda.min value which corresponded to 5 variables (age at diagnosis, sex, Ann Arbor stage, primary site, and year of diagnosis) (Fig. 3B and C). Univariate analyses demonstrated that age at diagnosis, marital status, Ann Arbor stage, primary site, number of bone lesions, therapy, and year of diagnosis were associated with DSS (*P* < .05, Table 4). In BESS, 8 variables (age at diagnosis, marital status, Ann Arbor stage, primary site, laterality, number of bone lesions, therapy, and year of diagnosis) were selected based on the maximum value of the adjusted R2 (Fig. 3E). Using LASSO regression, we selected lambda.min value which corresponded to 7 variables (age at diagnosis, sex, marital status, Ann Arbor stage, laterality, number of bone lesions, and year of diagnosis) (Fig. 3F and G).

**Table 4:** Univariable Cox regression analysis of OS and CSS for PB-DLBCL patients.

Variables	OS			CSS		
	HR (95% CI)		*P*	HR (95% CI)		*P*
Age	1.070 (1.061, 1.080)		<.001*	1.062 (1.050, 1.074)		<.001*
Race						
White		Reference			Reference	
Black	0.681 (0.412, 1.128)		.136	0.653 (0.334, 1.278)		.214
Other	0.923 (0.565, 1.506)		.748	0.895 (0.473, 1.694)		.733
Sex						
Male		Reference			Reference	
Female	0.974 (0.780, 1.218)		.820	0.987 (0.740, 1.317)		.929
Marital status						
Married		Reference			Reference	
Unmarried	0.503 (0.357, 0.710)		<.001^*^	0.624 (0.412, 0.944)		.026^*^
Ann Arbor stage						
Stage I		Reference			Reference	
Stage II	1.017 (0.701, 1.475)		.929	1.025 (0.622, 1.687)		.924
Stage III	0.907 (0.401, 2.052)		.815	0.834 (0.264, 2.639)		.757
Stage IV	1.446 (1.137, 1.840)		.003^*^	1.762 (1.297, 2.393)		<.001^*^
Primary site						
Axial		Reference			Reference	
Appendicular	0.566 (0.442, 0.726)		<.001^*^	0.591 (0.429, 0.815)		.001^*^
Craniofacial	0.615 (0.384, 0.985)		.043^*^	0.591 (0.319, 1.095)		.094
Laterality						
Bilateral, single primary		Reference			Reference	
Left: origin of primary	0.873 (0.274, 2.784)		.819	1.465 (0.201, 10.673)		.707
Right: origin of primary	1.308 (0.417, 4.101)		.645	2.213 (0.309, 15.857)		.429
Not a paired site	0.808 (0.253, 2.586)		.720	1.206 (0.164, 8.857)		.854
Number of bone lesions						
Single		Reference			Reference	
Multiple (⩾2)	1.839 (1.455, 2.324)		<.001*	1.383 (1.008, 1.899)		.045*
Surgery						
No		Reference			Reference	
Yes	1.209 (0.937, 1.561)		.144	1.141 (0.816, 1.596)		.441
Therapy						
Chemotherapy alone		Reference			Reference	
No therapy	1.474 (1.007, 2.157)		.046^*^	1.589 (0.972, 2.597)		.065
Chemotherapy + radiation	0.621 (0.479, 0.804)		<.001^*^	0.658 (0.471, 0.920)		.015^*^
Radiation alone	3.702 (2.614, 5.243)		<.001^*^	3.533 (2.267, 5.508)		<.001^*^
Year of diagnosis						
2000–2009		Reference			Reference	
2010–2018	0.814 (0.629, 1.053)		.117	0.663 (0.480, 0.916)		.013^*^

CI = confidence interval, DSS = disease-specific survival, HR = hazard ratio, OS = overall survival, PB-DLBCL = primary diffuse large B-cell lymphoma of bone.

After further backward stepwise multivariable Cox regression, 2 models (BESS and LASSO) were established for OS and DSS (the model based on univariate analyses was the same as that based on BESS). For OS, the model constructed with the variables screened by univariate regression and BESS had the smallest AIC of 3526.5, compared with 3548.8 for the LASSO regression. For DSS, the model constructed with the variables screened by univariate regression and BESS has the smallest AIC of 2202.5, compared with 2212.8 for the LASSO regression. An additional comparison of the AUC among the models was then performed (Fig. 3D and H). Finally, age at diagnosis, Ann Arbor stage, primary site, and therapy were selected as independent predictors to develop a predictive model for OS. Age at diagnosis, marital status, Ann Arbor stage, number of bone lesions, therapy and year of diagnosis were selected as independent predictors to develop the predictive model of DSS.

### 3.3. Nomogram construction and classical application of the nomogram

**Figure 4.**
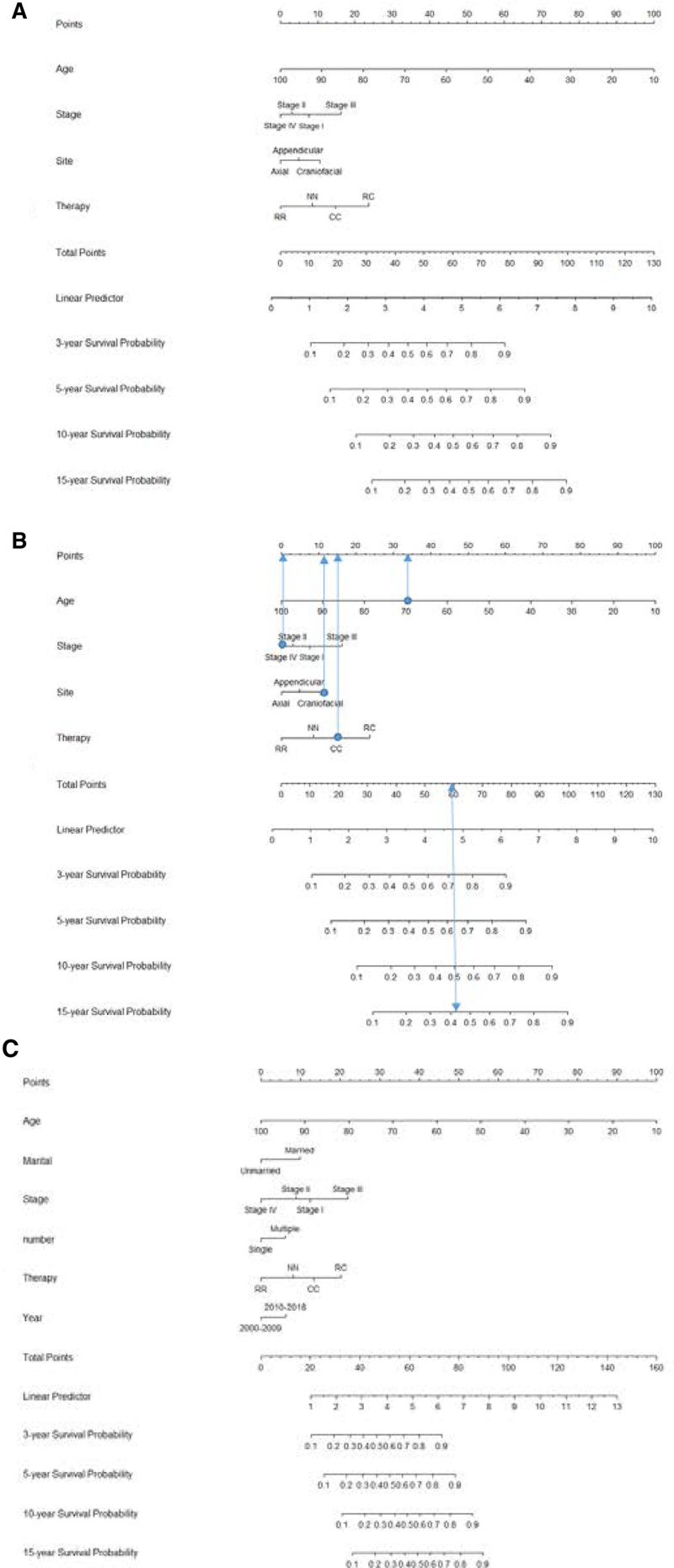
Nomogram for 3-, 5-, 10- and 15-year prediction of OS and DSS of patients with PB-DLBCL. (A) Nomogram for 3-, 5-, 10- and 15-year prediction of OS (B) Each category of the prognostic variables was assigned a score based on the points scale. After summing up the score of each variable and locating the total score on the total points scale, a line was vertically drawn to the 3-, 5-, 10- and 15-year survival probability scale and estimated survival probability could be obtained (C) Nomogram for 3-, 5-, 10- and 15-year prediction of DSS. CC = chemotherapy alone, NN = no therapy, RC = chemotherapy + radiation, RR = radiation alone.

Independent predictors were used to establish nomograms to estimate the probability of 3-, 5-, 10-, and 15-year OS and DSS (Fig. 4A and C). The survival outcomes of patients with PB-DLBCL were predicted by calculating the total score. How to use the nomogram was presented in Figure 4B. The patient was a 70-year-old male, who was diagnosed with stage IV PB-DLBCL. He had craniofacial bone lesions and received initial chemotherapy. In Figure 4B, vertical lines was drawn from each factor to the point score. Therefore, he scored 33 points for the age of 70, 0 points for stage IV, 11 points for craniofacial bone lesions and 15 points for receiving chemotherapy. Then summing up the point score, from “Total points” a line was drawn straight down to each survival probability axis. He received a total score of 59 points, and the survival probability was predicted to be 72% at 3 year, 63% at 5 year, 50% at 10-year, and 42% at 15year.

### 3.4. Nomogram performance

**Figure 5.**
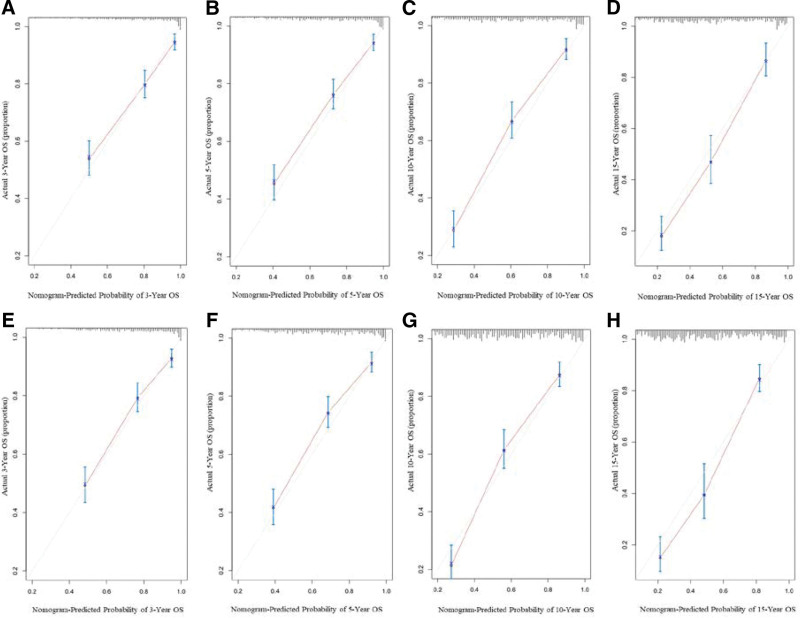
Calibration curves for predicting 3-, 5-, 10-, and 15-year OS of patients with PB-DLBCL in training set A, B, C, D and validation set E, F, G, H. The nomogram-predicted probability of OS was plotted on the x-axis, with actual OS plotted on the y-axis. The calibration curves were visual representations of the relationship between the predicted and actual absolute risk.

**Figure 6.**
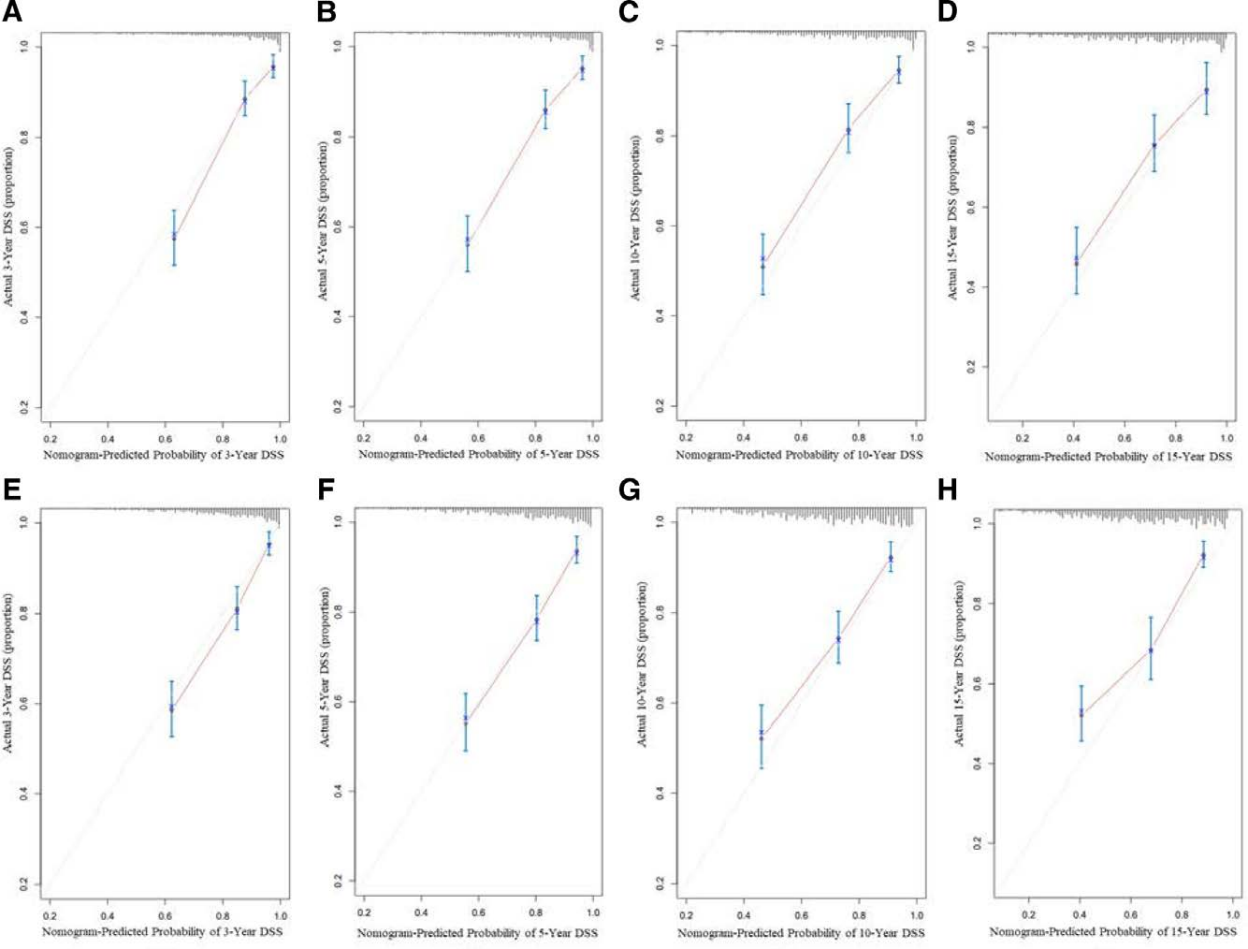
Calibration curves for predicting 3-, 5-, 10-, and 15-year DSS of patients with PB-DLBCL in training set A, B, C, D and validation set E, F, G, H. The nomogram-predicted probability of DSS was plotted on the x-axis, with actual DSS plotted on the y-axis. The calibration curves were visual representations of the relationship between the predicted and actual absolute risk.

**Figure 7.**
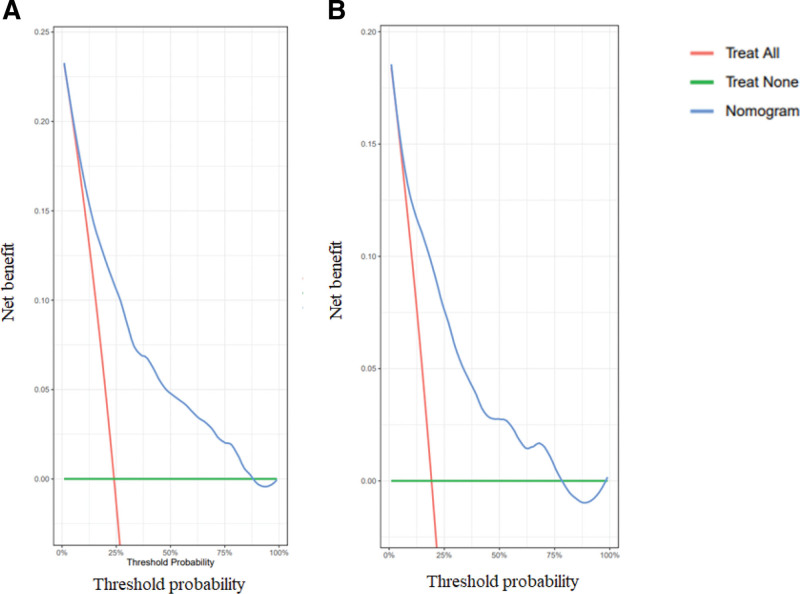
DCA plots of the nomogram for 3-year survival prediction in the training set A (OS) and B (DSS). DCA plot depicted the model with superior clinical application.

In our study, the nomogram was used for both internal and external validations. In the training set, the C-index of the developed nomograms for predicting OS and DSS were 0.784 (95% CI, 0.758–0.810) and 0.785 (95% CI, 0.753–0.817), respectively. In the validation set, the C-index of the nomograms for predicting OS and DSS were 0.759 (95% CI, 0.733–0.785) and 0.752 (95% CI, 0.719–0.785), respectively. The calibration plots demonstrated superb consistency between the nomogram-predicted survival probability and actual observation (Figs. 5 and 6). DCA plots for the established nomograms were presented in Figure 7. These results demonstrate that the predictive nomogram has high net benefits and good clinical utility.

## 4. Discussion

PB-DLBCL is a rare extra-nodal lymphoma. Few related studies have been reported, and they were mainly single-center studies with limited sample sizes. Therefore, the clinical characteristics, treatment, and prognosis of PB-DLBCL are currently ambiguous. Patients with the same tumor may have different clinical outcomes owing to multiple significant prognostic factors.^[17]^ Nomogram is a practical tool to quantify risks and calculate the probability of survival outcome. In our study, patients aged < 18 years were excluded because of their different natural disease histories. Our study analyzed 3112 patients with PBL in the SEER database, which 2101 (67.51%) patients was be with PB-DLBCL. Compared with the previous studies (66.58% to 80%),^[5,7]^ our study achieved consistent conclusions. After DLBCL, follicular lymphoma is the second most common pathological type in PBL.^7^ However, a Chinese study found that histological subtype of T-cell lymphomas are second only to DLBCL.^[18]^ Overall 1603 adult patients with PB-DLBCL were included in our study, which is the largest adult cohort to date. Additional demographic and clinical variables were included. In addition, the tumor sites were more refined and was divided into axial, appendicular, and craniofacial. Based on clinical data, some studies suggested that patients with PB-DLBCL have a good prognosis and long survival time. The 5-year survival rate of PB-DLBCL patients usually exceeded 70% from previous studies.^[4,19]^ In our study, the 5-year OS rate was consistent with that of previous studies, and the 5-, 10-, and 15-year OS rates of adult patients with PB-DLBCL were 70.5%, 60.0%, and 49.9%, respectively. However, few studies did not suggest such a good prognosis of PB-DLBCL. Zhang et al^[18]^ reported the 5-year OS of 34 PB-DLBCL cases were 53%. Considering the regional differences and variations in treatment and selection biases in retrospective studies, it may not be surprising that there are significant differences in the results between independent studies. Compared with other studies, our study also provided the 10- and 15-year survival rates of patients with PB-DLBCL in order to provide reference for clinicians. A total of 400 deaths occurred during the 3-years follow-up period, 79.00% of patients died of PB-DLBCL and 21.00% died of other causes. A total of 230 patients have died between 3 and 15 year timeline, 29.13% of patients died of PB-DLBCL and 70.87% died of other causes. The non-relapse mortality is high which is due to diseases of heart (28.83%) and second malignancy (17.79%). Diseases of heart maybe related to cardiac toxicity caused by doxorubicin.^[20]^ It would be an important pointer that treatment should be de-escalated in some patients (esp. good responders).

Univariate analyses demonstrated that only age, Ann Arbor stage, marital status, primary site, number of bone lesions, radiation and chemotherapy were associated with OS. Sex was not a predictive factor in the univariate assessment. The male-to-female ratio was 1.15:1, which is based on the largest sample size in our study. Some retrospective studies with small samples mentioned that PB-DLBCL have a similar incidence in men and women.^[9,21]^ To ensure the model’s goodness-of-fit, we used 3 methods to screen for prognostic factors. Ultimately, age, Ann Arbor stage, primary site, and therapy were confirmed as independent predictors of OS to develop the nomogram, while age, marital status, Ann Arbor stage, number of bone lesions, therapy, and year of diagnosis were confirmed as independent predictors of DSS. In nomograms, age played a major role in the scoring system. Age is a significant predictor for OS and DSS.^[10]^ He-Hui Wang et al reported that PBL patients with age more than 75 years and patients between 61 and 75 have 2 to 7 times risk of death for DSS and OS, compared to patients with age <60 years.^[22]^ Older patients usually had number of comorbidities and exhibited poor physical status, which may increase risk of morbidity and mortality. Most patients (54.1%) had stage I and 31.5% had stage IV in our study. A previous study showed that a higher Ann Arbor stage was an unfavorable factor for patients with PBL,^[7]^ and our study achieved consistent conclusions for patients with PB-DLBCL. One study noted that the survival of PBL patients with involved axial skeletons was significantly lower than that of those with appendicular and craniofacial skeletons.^[7]^ This could be related as paraplegia may affect performance status, thereby impacting survival.^[4]^ Fracture is an independent risk factor in PB-DLBCL patients.^[23]^ Pathological fracture is 1 of the most common complications of bone lymphomas, varying in frequency between 10% and 20%.^[23]^ The incidence of a second fracture being 10% after anti-lymphoma treatment.^[24]^

PB-DLBCL is a rare bone malignancy for which clear therapeutic guidelines are lacking. Therapy affects patient survival. In the nomograms, patients who received combination of chemotherapy and radiation yielded the highest score, and those who received radiotherapy alone yielded the lowest score. Currently, CHOP and CHOP-like regimens, with or without radiotherapy, are the preferred treatments for patients with PB-DLBCL. The IELSG-14 study showed that PB-DLBCL patients with stage I–II had a good prognosis treated with CHOP chemotherapy followed by radiotherapy or not.^[4]^ The modified BFM-90 showed high efficacy and safety in 23 patients with advanced-stage PB-DLBCL.^[25]^ Twelve (52%) of these patients received rituximab prior to therapy.^[26]^ After 2000, rituximab was added to CHOP-like chemotherapy for PB-DLCBL, which had significantly improved the survival outcome of PB-DLCBL patients.^[11]^ Surgical treatment was usually used when patients had pathological fractures, symptoms of compression, or biopsy. In patients with local recurrence, extensive excision and reconstruction can improve the prognosis of PBL patients.^[26]^

The value of radiotherapy for management of PB-DLCBL is still controversial.^[13,14]^ In the 1960s, local radiotherapy had been established as an effective local treatment for PBL with locoregional recurrence rates of 10% to 20%. However, the distant recurrence rate was ~50% and the 5-year OS rate was only 55% to 65%.^[27,28]^ The combination of chemotherapy and radiotherapy improved the 5-year OS rate to approximately 70% to 90%.^[3,29,30]^ The IELSG-14 confirmed that PB-DLBCL patients treated with local radiotherapy, whether followed by chemotherapy or not, had a worse survival outcome than those treated with chemotherapy, whether followed by radiotherapy or not.^[4]^ Moreover, adding radiotherapy after chemotherapy did not improve the prognosis of patients.^[4]^ Another study had shown that the order of radiotherapy and chemotherapy could also affect patient outcomes. Seventy-eight PB-DLBCL patients with pathological fractures managed with chemotherapy followed by irradiation had better survival outcomes than patients treated with the inverse sequence.^[24]^ In addition, a new research revealed that radiotherapy addition may be beneficial especially in patients with PBL in partial response after 4 cycles of chemotherapy.^[31]^

Compared to PB-DLBCL patients without radiotherapy, patients treated with radiotherapy had a better survival outcome, based on 1654 patients with PB-DLBCL from 1973 to 2010. After 2000, rituximab was added to the chemotherapy regimen for the treatment of PB-DLCBL. The investigator take year 2000 as the cutoff point for sub-analysis. For PB-DLBCL patients diagnosed before 2000 (n = 517), those who received radiotherapy (n = 367) had a significantly better survival than those who did not. However, for PB-DLBCL patients diagnosed after 2000 (n = 1037), there were no differences in survival outcomes between patients receiving and not receiving radiotherapy.^[32]^ With the awareness of the side effects of radiotherapy, such as a higher rate of second primary malignancies,^[33]^ the concept of omitting the use of radiotherapy has been proposed in recent years. Early- and advanced-stage patients with PB-DLBCL were frequently analyzed together in previous studies. Ma et al identified that early stage (stage I–II) patients could benefit from radiotherapy in the rituximab era by sub-analysis, but radiotherapy is not associated with better outcomes in patients with advanced-stage disease.^[34]^

Our study has certain limitations. First, the SEER database can’t provide important information on disease progression, comorbidities, and complications. Some variables associated with prognosis are unavailable in the SEER dataset, such as fracture, rituximab use, local relapse, and detailed regimens of radiation doses and chemotherapy agents. External validation was not performed using information from other databases. Despite these limitations, independent prognostic factors for survival were identified for PB-DLCBL patients and nomograms were established to predict the OS and DSS of PB-DLBCL patients, which could help clinicians make scientific decisions.

## 5. Conclusions

Based on the SEER database, the most frequent pathological type of PBL in adults is DLBCL, accounting for 67.51%.The most common sites involved in PB-DLBCL are the spine, followed by the long bones of the lower limbs. Three methods were used to screen for prognostic factors. Ultimately, age, Ann Arbor stage, primary site and therapy were confirmed as independent predictors of OS to develop the nomogram in adult patients with PB-DLBCL. Age, marital status, Ann Arbor stage, number of bone lesions, therapy and year of diagnosis were confirmed as independent predictors of DSS to develop the nomogram. Chemotherapy played an important role in the PB-DLBCL treatment. Two nomograms were developed to predict the OS and DSS of patients with PB-DLBCL, and they showed favorable applicability and accuracy. It will enable accurate prognosis predictions for adult patients with PB-DLBCL and help clinicians make scientific decisions.

## Author contributions

**Conceptualization:** Donghua Liu.

**Formal analysis:** Xiangpeng Li.

**Supervision:** Changkai Zhou.

**Validation:** Tong Gao, Qie Guo.

**Writing – review & editing:** Jing Li.
